# A First in Human Clinical Trial Assessing the Safety and Immunogenicity of Two Intradermally Delivered Enterotoxigenic *Escherichia coli* CFA/I Fimbrial Tip Adhesin Antigens with and without Heat-Labile Enterotoxin with Mutation LT(R192G)

**DOI:** 10.3390/microorganisms11112689

**Published:** 2023-11-02

**Authors:** Ramiro L. Gutiérrez, Mark S. Riddle, Chad K. Porter, Milton Maciel, Steven T. Poole, Renee M. Laird, Michelle Lane, George W. Turiansky, Abel Jarell, Stephen J. Savarino

**Affiliations:** 1 Naval Medical Research Command, Silver Spring, MD 20910, USA; rlgm72@gmail.com (R.L.G.); spoole102@gmail.com (S.T.P.);; 2 Uniformed Services University of the Health Sciences, Bethesda, MD 20814, USA; 3 Henry M. Jackson Foundation for the Advancement of Military Medicine, Inc., Bethesda, MD 20817, USA; 4 Walter Reed National Military Medical Center, Bethesda, MD 20814, USA

**Keywords:** enterotoxigenic *E. coli*, intradermal, vaccine, immunogenicity

## Abstract

Introduction: Enterotoxigenic *E. coli* (ETEC) is a leading cause of diarrhea in travelers as well as for children living in low- to middle-income countries. ETEC adhere to intestinal epithelium via colonization factors (CFs). CFA/I, a common CF, is composed of a polymeric stalk and a tip-localized minor adhesive subunit, CfaE. Vaccine delivery by the transcutaneous immunization of dscCfaE was safe but was poorly immunogenic in a phase 1 trial when administered to volunteers with LTR(192G) and mLT. To potentially enhance the immunogenicity of CfaE while still delivering via a cutaneous route, we evaluated the safety and immunogenicity of two CfaE constructs administered intradermally (ID) with or without mLT. Methods: CfaE was evaluated as a donor strand-complemented construct (dscCfaE) and as a chimeric construct (Chimera) in which dscCfaE replaces the A1 domain of the cholera toxin A subunit and assembles non-covalently with the pentamer of heat-labile toxin B (LTB). Subjects received three ID vaccinations three weeks apart with either dscCfaE (1, 5, and 25 µg) or Chimera (2.6 and 12.9 µg) with and without 0.1 µg of mLT. Subjects were monitored for local and systemic adverse events. Immunogenicity was evaluated by serum and antibody-secreting cell (ASC) responses. Results. The vaccine was well-tolerated with predominantly mild and moderate local vaccine site reactions characterized by erythema, induration and post-inflammatory hyperpigmentation. High rates of serologic and ASC responses were seen across study groups with the most robust responses observed in subjects receiving 25 µg of dscCfaE with 0.1 mcg of LT(R192G). Conclusion: Both ETEC adhesin vaccine prototypes were safe and immunogenic when co-administered with mLT by the ID route. The observed immune responses induced with the high dose of dscCfaE and mLT warrant further assessment in a controlled human infection model.

## 1. Introduction

Diarrheal diseases are a leading cause of morbidity and mortality worldwide with the greatest burden in children living in low- to middle-income countries (LMICs) [1,2,3,4]. Among the most common causes of infectious diarrhea in these populations is enterotoxigenic *E. coli* (ETEC) [5,6,7,8,9,10]. While ETEC-attributable mortality estimates vary greatly [11], prospective case-control and cohort studies repeatedly identify ETEC as a leading cause of diarrhea with a spectrum of illness ranging from mild to severe [10,12,13]. Additionally, non-fatal infections with ETEC have long-lasting effects and contribute to stunting and deaths due to other infectious diseases, further highlighting the morbidity of ETEC in LMIC pediatric populations [14].

In addition to LMIC pediatric populations, ETEC has been repeatedly identified as the leading cause of travelers’ diarrhea (TD) among travelers from high-income countries (HICs) to LMICs [15]. Among civilian travelers, ETEC is estimated to account for approximately one-third of all TD cases with comparable estimates across South America, the Caribbean, Africa, and South Asia [16]. Similarly, among military populations, a healthy subset of the general travel population, ETEC is a leading cause of TD globally [15,17]. Severity in ETEC-attributable TD ranges from a mild illness with limited impact to travel to a cholera-like purging requiring antibiotic treatment, cancellation of travel plans, and duty re-assignments [18,19,20].

ETEC adhere to the small intestine via colonization factors (CFs) [21]. Attachment, followed by the production of a heat-labile (LT) and/or heat-stable (ST) enterotoxin, results in secretory diarrhea and symptoms of variable severity. All ETEC vaccines under development, including live-attenuated, inactivated whole-cell, and subunit approaches target the CFs and LT either alone or in combination [22]. One of the most globally prevalent CFs is CFA/I, and it has been a focus of prototype subunit vaccine approaches. Upon the elucidation of the CFA/I subunit structure, we developed an adhesin-based ETEC vaccine approach which may enable broader in-class cross-CF protection while also minimizing valency requirements [23,24]. Passive and active protection based on anti-CfaE, antibodies against the adhesin subunit of CFA/I, has been demonstrated in humans and non-human primates [25,26,27]. Similarly, it is likely that anti-LT responses will also be necessary; thus, LTR(192G), mLT, was co-administered with the CF antigens due to its adjuvant capacity with co-administered antigens [28]. Herein, we expand on our prior evaluation of donor-strand-complemented CfaE (dscCfaE) administered transcutaneously (TCI) with mLT by evaluating the intradermal (ID) route of vaccination. We also evaluated a chimeric form of CfaE (Chimera), a construct in which dscCfaE replaces the A1 domain of the cholera toxin A subunit and assembles non-covalently with the pentamer of heat-labile toxin B (LTB) [29]. We co-administered each dscCfaE antigen with mLT, which has been shown to help potentiate mucosal responses [30,31]. We sought to evaluate the adjuvanticity properties when administered by the ID route. This trial represents the first attempt at the intradermal administration of mLT in humans.

Although an immune correlate of protection for ETEC has not been established, data from human and animal studies suggest serologic responses against colonization factor antigens and LT-toxins are associated with protection [28,32,33,34,35,36]. It is hypothesized that an effective ETEC vaccine will need to generate a sufficient immune response to the targeted antigens at the small intestinal mucosa. While the oral delivery of vaccines has been shown to efficiently induce such mucosal responses, there is growing concern about the lesser efficacy of such vaccines amongst some populations in the developing world [37]. We previously demonstrated that TCI-administered dscCfaE, with mLT, induced functional fecal and serum responses in mice, suggesting the existence of a skin–gut axis of immune response [38]. Although a similar mucosal immune response following cutaneous vaccination has yet to be fully described in humans, prior work suggests it may exist [34]. From an vaccination route standpoint, the use of ID and transcutaneous immunization (TCI) may offer an advantage if able to elicit responses against mucosal pathogens at the site of infection [39].

## 2. Materials and Methods

### 2.1. Clinical Trial Design

This was an open-label, phase 1 clinical trial in which 49 subjects were scheduled to receive dscCfaE or Chimera, with or without 0.1 µg of mLT, by the ID route in a dose-escalating design (Table 1). A group receiving 1250 µg of dscCfaE co-administered with 50 µg mLT transcutaneously was also included; however, those data are reported elsewhere [29]. The initial cohort consisted of 3 groups of 5 subjects receiving dscCfaE (1 µg), Chimera (2.6 µg) or mLT (0.1 µg) alone. The dscCfaE doses in the two subsequent cohorts were 1 µg and 5 µg, each in combination with mLT (0.1 µg). The Chimera doses were the molar-matched equivalent to the dscCfaE doses: 2.6 µg and 12.9 µg. In the final cohort, 10 subjects received 25 µg of dscCfaE, co-administered with mLT (0.1 µg), and 8 subjects received 1250 µg of dscCfaE via the TCI route co-administered with 50 µg mLT. All vaccinations were administered to alternating deltoid regions on Days 0, 21 and 42. Each subject received the same dose at each vaccination visit per their group assignment. The study followed the same assessment methods and schedule as was used in the TCI evaluation of safety and immunogenicity of dscCfaE and mLT [29].

The primary objective was to evaluate the safety and tolerability of dscCfaE and Chimera with and without mLT when administered ID to healthy adults. Throughout the trial, an independent safety monitoring committee reviewed all safety data prior to the advancement of each cohort. The secondary objective was to evaluate immune responses to the immunizing antigens. The study was approved by the Naval Medical Research Command and Walter Reed Army Institute of Research institutional review boards and was registered in ClinicalTrials.gov (NCT01644565).

### 2.2. Study Population and Enrollment Criteria

Volunteers were healthy male and non-pregnant female adults between 18 and 45 years of age recruited from the greater Washington, DC metropolitan area and were enrolled after an informed consent process consisting of a detailed presentation of study material via a taped video, successful completion of a comprehension test, and interview with an investigator. Volunteers were excluded from enrollment if they had clinically significant acute or chronic diseases; immunosuppressive disorders or medication; regularly used antidiarrheal, anti-constipation, or antacid therapy; reported an abnormal stool pattern (<3 stools per week, or >3 stools per day); had participated in other investigational product research; had a positive blood test for Hepatitis B surface antigen, Hepatitis C virus, or Human Immunodeficiency Virus-1; or had clinically significant abnormalities on basic laboratory screening. Furthermore, given the intradermal route of administration, volunteers were excluded if they had current acute skin infections, active irritant or contact dermatitis, a past/current medical history of chronic skin disorders, or history of atopy. Lastly, volunteers with a history of microbiologically confirmed ETEC or *V. cholera* infection, prior receipt of an experimental ETEC or *V. cholera* vaccine, recent travel (≤2 years) to a country where ETEC or *V. cholera* or other enteric infections are endemic, or recent (≤3 years) occupation involving exposure to ETEC or *V. cholera* were excluded.

### 2.3. Manufacture of dscCfaE, Chimera and mLT Vaccine Components

The investigational vaccine components, dscCfaE, Chimera and mLT, were manufactured using current Good Manufacturing Practices (cGMPs). The dscCfaE component is a recombinant protein purified from a host *E. coli* expression strain and has been previously described [23]. A cGMP lot of purified bulk dscCfaE was manufactured at the WRAIR Pilot Bioproduction facility (PBF) and filled in 0.7 mL aliquots with a concentration of 3.43 mg/mL, 20 mM sodium phosphate and 200 mM sodium chloride at a pH of 6.2, into 3 mL depyrogenated glass vials which were stoppered, crimped and stored at −80 °C. The mLT, produced from the recombinant *E. coli* strain JM83 (pLC326), was constructed at Tulane University in the laboratory of John Clements [40]. mLT was manufactured under cGMP at the WRAIR PBF. The final product was lyophilized in vials containing 1 mg of protein per vial.

The Chimera is a recombinant protein purified from a host *E. coli* expression strain and is encoded by two genes, a *dsc_14_cfaE-ctA2* fusion gene and a mutant *ltB* gene [41]. The *dsc_14_cfaE-ctA2* gene encodes a donor strand-complemented form of CfaE, which has a truncated form of the A2 domain of the Cholera toxin A subunit, sCTA2, fused to its C-terminus. Donor strand complementation stabilizes CfaE by C-terminal fusion with the first 14 amino acids (dsc_14_) of CfaB, which is the major structural subunit of CFA/I. The mutant *ltB* gene is a hybrid gene cloned from a wild-type ETEC strain, H10407, and a Throop D lab strain that encodes the B subunit of a type I heat-labile enterotoxin containing two histidine amino acid substitutions at positions 13 (R13H) and 94 (N94H). LTB monomers form a pentameric ‘ring’ structure, and the dsc_14_CfaE-sCTA2 fusion protein assembles with the LTB pentamer through the sCTA2, which extends through a central pore in the LTB pentamer forming a shape that resembles a ‘finger in a ring’. The final chimera is therefore a non-covalently assembled hetero-hexamer composed of a single copy of the dsc_14_CfaE-sCTA2 assembled with five copies of the LTB subunit. The Chimera was produced by the WRAIR PBF under cGMP and is stored in vials containing a 0.7 mL aqueous solution with 3.50 mg/mL Chimera protein in 20 mM sodium phosphate and 135 mM sodium chloride.

### 2.4. Vaccine Formulation

On the days of vaccination, bulk doses were made according to the study group. Briefly, the mLT was reconstituted with sterile water and diluted with sterile saline to the target concentration. The dscCfaE and Chimera were thawed and diluted with sterile saline to the target doses and admixed with the diluted mLT. The bulk was stored at 2–8 °C until prepared for subject vaccination. Once an eligible subject had completed a medical history review and eligibility had been verified, unblinded staff drew up 0.1 mL of the appropriate vaccine into a syringe labeled with the subject’s identification number and provided the syringe to the clinician. All doses were administered within 6 h of formulation, and all formulation procedures were witnessed and verified by an independent researcher.

### 2.5. Immunization Procedures

After assessing continued eligibility, the vaccination site was cleaned with an alcohol swab. The vaccine dose was drawn into a standard tuberculin syringe with a 27-gauge beveled needle by the pharmacy staff and provided to the vaccinating clinician. The vaccine was administered intradermally using a standard Mantoux injection to the deltoid. All vaccinations were administered by the same trained clinician.

Subjects were observed for ≥30 min post-vaccination, and vital signs were collected. Diary cards were given to record local and systemic reactions for 7 days after each dose. Subjects were provided with a transparent overlay to facilitate the assessment of local site reaction circumference. Subjects returned approximately 24 h after application for an assessment by a clinician.

### 2.6. Safety Assessment

Adverse event monitoring, which began after the first vaccination and ended 28 days after the third vaccine dose, was conducted using in-person symptom surveillance, symptom diary log, and targeted physical exams. For a week after each vaccination, volunteers documented all adverse events in a diary and reviewed entries with a clinician at the Day 2 and 7 visits, where they also underwent a focused clinical exam. Solicited systemic adverse events included malaise, headache, joint aches, muscle aches, loose stools and fever. Solicited local site findings included vaccine site reactions (rash), itching, pain, and tenderness. In addition to the self-reporting of vaccine site reactions, standardized history and physical examinations with visualization of the vaccine sites were conducted by trained physicians using a standardized vaccine site assessment grading scale which was developed under the guidance of Board Certified dermatologists who also served as co-investigators in the trial [42]. The local examination of vaccine sites included an assessment for erythema, papules/plaques, pigmentation changes, edema, vesicles/bullae, and ecchymosis. Ulcerations or erosions were specifically sought and recorded if present. The severity of self-reported symptoms was assessed according to the following scale: absent, mild (not interfering with routine activities), moderate (interfering with daily activities), and severe (preventing routine activities). Furthermore, blood to assess hematological and serum chemistry changes was drawn seven days following each vaccine dose. The relationship of an adverse event to the vaccine was determined by the principal investigator. All aspects of the trial were closely assessed by an independent medical monitor and the SMC.

### 2.7. Immunological Assessments

Specimens were collected prior to and during the vaccination period and used for immunological analysis. Serum was assessed for antigen-specific (dscCfaE and LTB) IgA and IgG responses, and peripheral blood mononuclear cells (PBMCs) were assessed for antibody-secreting cells (ASCs). PBMCs were obtained using a Ficoll–Hypaque gradient technique evaluated for antigen-specific ASCs on the same day of collection. Additional exploratory immunological assessment conducted included hemagglutination inhibition assays to detect functional neutralizing antibody responses in serum.

#### 2.7.1. Antigen-Specific ELISA

Subjects’ sera were evaluated for IgG and IgA anti-dscCfaE and -LTB antibody (Ab) titers by ELISA. IgG antigen (Ag)-specific ELISA was performed on Nunc™ MicroWell™ 96-well plates, while IgA Ag-specific assay was performed on Nunc™ MicroWell™ Maxisorb™ (Thermo Scientific, Rochester, NY, USA) 96-well plates. For anti-dscCfaE-specific assays, plates were coated with dscCfaE at 1 µg/mL, while plates for LTB-specific assays were first coated with GM1 (Sigma-Aldrich, Saint Louis, MO, USA) at 0.5 µg/mL, both with 100 µL/well, for 1 h at 37 °C, followed by overnight (O/N) at 4 °C. All plates were blocked with 200 μL/well of 5% non-fat milk (Sigma-Aldrich) in 0.05% Tween-20 (Sigma-Aldrich)-PBS (PBS-T) for 60 min at 37 °C in a humidified chamber. LTB was added to LTB-specific plates at 0.5 µg/mL and incubated at 37 °C for 1 h. After three washes with PBS-T, serum samples were added at a starting dilution of 1:50 in 1% (for IgG) or 2% (for IgA) non-fat milk-PBS-T followed by a 3-fold serial dilution and incubated for 1.5 h at 37 °C in a humidified chamber. Plates were washed 5 times with PBS-T followed by the addition of 0.5 µg/mL peroxidase-conjugated goat anti-human IgG (KPL, Gaithersburg, MD, USA) or 0.25 µg/mL biotin-conjugated anti-human IgA (KPL) in 1% (for IgG) or 2% (for IgA) non-fat milk-PBS-T for 1.5 h at room temperature (RT). For IgA assays, plates were washed and ExtrAvidin^®^-Peroxidase (Sigma-Aldrich) was added at 1:2000 for 30 min at RT. After final washes, 2,2′-azino-bis(3-ethylbenzothiazoline-6-sulfonic acid (ABTS) (KPL) or 3,3′,5,5′-tetramethylbenzidine (Ultra-TMB) (Thermo Scientific, Waltham, MA, USA) was added to IgG or IgA assays, respectively, according to the manufacturer’s recommendations. After 30 min incubation, optical density (OD) was measured at 450 or 405 nm for ABTS or Ultra-TMB, respectively, using a Multiskan EX^®^ ELISA reader with Ascent^®^ software (Thermo Scientific), which calculated the final Ab titers. The cutoff for each plate was calculated by the average of the background wells OD plus a fixed value of 0.4. A linear regression was fitted to the experimental data, and the endpoint titer was determined as the reciprocal of the interpolated sample dilution that intersected with the cutoff and log_10_-transformed. All pre- and post-vaccination samples from a given subject were assayed side by side on the same plate, and each sample was tested in duplicate. The average log_10_ titer for the duplicates was calculated as the final result. Serum samples with OD below the cutoff, even at the top serum dilution, were assigned a value of one-half of the lower detection limit (i.e., 1:50) for computational purposes. For interpretation purposes, anti-LTB responses are proxy to anti-toxin responses.

#### 2.7.2. Hemagglutination Inhibition Assay (HAI)

The levels of ETEC-specific functional blocking antibodies were evaluated by hemagglutination inhibition (HAI) assays, which is an adaptation of the mannose-resistant hemagglutination assay (MRHA) [43]. Bovine red blood cells (BRBCs) were purchased from Lampire Laboratories (Pipersville, PA) and stored for up to 10 days at 4 °C in Alsever’s solution prior to use. In order to guarantee the day-to-day reproducibility of the HAI assay, for each assay day, we measured the Minimum Hemagglutination Titer (MHT), i.e., the highest dilution of bacteria that caused the complete agglutination of BRBC. Briefly, CFA/I^+^ ETEC bacteria (strain H10407) were grown O/N on CFA agar plates with bile salts, harvested, and resuspended in 0.5% D-mannose (Sigma) in PBS (PBS-M) to a final solution with OD_650_ 0.2 ± 0.02. Then, 25 µL of this suspension was added to wells of microtiter plates (Falcon Microtest™ U-bottom tissue culture treated, BD, Franklin Lakes, NJ, USA), which was followed by 2-fold serial dilution with PBS-M. Afterwards, 25 µL of 1.5% BRBC in PBS-M plus 25 µL of PBS-M was added to each dilution, plates were shaken at 500 RPM for 30 min at 4 °C, and agglutination was recorded. The highest dilution to give visible agglutination was taken as the MHT for the day. The HAI assay was performed with a bacteria suspension two dilutions more concentrated than the one defined as MHT. For the HAI assay, 25 µL of the serum samples was diluted 1:8 with PBS-M and added to the wells, which was followed by 2-fold serial dilution (up to 1:16,384) in PBS-M. The appropriate suspension of bacteria (according to MHT) was added in equal volume, and the plate was shaken at 500 RPM for 30 min at RT. After the incubation, 25 µL of 1.5% BRBC were added/well, the plate was shaken at 500 RPM for 30 min at 4 °C, and the presence or absence of agglutination recorded immediately after. Serum samples showing agglutination inhibition at 1:16,384 were re-assayed with higher dilutions. Each sample was tested in duplicate, and the HAI titer was expressed as the average of the reciprocal of the highest serum dilution that completely inhibited MRHA.

#### 2.7.3. ELISPOT

Ag-specific IgG and IgA antibody-secreting cells (ASCs) were enumerated by enzyme-linked immunosorbent spot (ELISPOT) assays. Briefly, 96-well ELISPOT MAHA (Millipore, Billerica, MA, USA) plates were coated in triplicate with dscCfaE at 2 µg/mL, GM1 (Sigma-Aldrich) at 5 µg/mL, or PBS only (negative control wells) O/N at 4 °C. After three washes with PBS, wells were blocked with complete RPMI (cRPMI; RPMI (Invitrogen, Carlsbad, CA, USA) plus 10% fetal calf serum (FCS) (ATCC, Manassas, VA, USA), 1% penicillin (10,000 IU/mL)/streptomycin (10,000 µg/mL) (Gibco Lab Inc., Grand Island, NY, USA), and 1% glutamine (Gibco)) for at least 1 h at RT. GM1-coated wells were then washed three times with PBS, and LTB was added at 5 µg/mL for approximately 2 h at RT. Prior to the addition of cells, all wells were washed three times with PBS and then filled with 100 µL of cRPMI. Then, 100 µL of freshly isolated PBMCs at 5 to 10 × 10^6^/mL was added per well, in triplicate, and incubated O/N at 37 °C and 5% CO_2_. Plates were washed three times with PBS-T, three min soaking in between, which was followed by two washes with PBS, and then they were incubated with 100 µL of 0.5 µg/mL of Biotin-SP-AffiniPure™ goat anti-human IgG, Fcγ fragment specific (Jackson ImmunoResearch Labs, West Grove, PA, USA) or Biotin-SP-AffiniPure goat anti-human serum IgA, α-chain specific (Jackson ImmunoResearch Labs) diluted in 0.5% BSA (Sigma-Aldrich) in PBS (PBS-BSA) for approximately 2 h at RT. After washing with PBS-T and PBS as previously described, 100 µL/well of ExtrAvidin^®^-Peroxidase (Sigma-Aldrich) diluted 1:1500 in PBS-BSA was added to wells and incubated for 1 h at RT. After further washes, the substrate 3-Amino-9 ethylcarbazole (AEC; Calbiochem, La Jolla, CA, USA) was added at 100 μL per well for approximately 15 min at RT, and the reaction was stopped with dH_2_O. The final enumeration of Ag-specific ASCs was performed using the ImmunoSpot Series 6 micro analyzer ELISPOT reader with aid of the Immunospot software version 5.1 (Cellular Technologies Ltd., Shaker Heights, OH, USA). Results were calculated by subtracting the average of spots counted in Ag-specific wells from negative control wells and presented as the number of Ag-specific ASC/10^6^ PBMC.

### 2.8. Study Endpoints and Definitions

The primary safety outcome was local or systemic adverse events 7 days post-vaccination. A priori immunological outcomes for responders were established as follows: seroconversion: ≥4-fold increase over the baseline titer; ASC response: ≥-fold increase over baseline number of ASC per 10^6^ PBMCs or post-vaccination value >1 ASC per 10^6^ PBMCs if baseline was 0.

### 2.9. Data Analysis and Statistical Considerations

Rates of all adverse events (related and unrelated) observed during the follow-up period after vaccinations were analyzed to compare within and across dose levels. For immunological analysis, qualitative (responder rates) and quantitative assessments (log transformed values) were made in addition to evaluation of the kinetics of the immune response. Non-parametric tests were used for comparison between groups (Kruskal–Wallis for continuous data and Fisher’s exact test for categorical data). Paired tests were used to compare individual post-vaccination values to baseline within each treatment group. All statistical tests were interpreted in a two-tailed fashion using alpha = 0.05; no adjustments were made for multiple comparisons.

As this was a phase 1 dose-finding clinical trial, the sample size for this study was designed to evaluate preliminary safety data and not designed to show statistical differences between cohorts. Furthermore, given the small number of subjects per group, the precision of our estimate for adverse events is limited. For example, using binomial probability formulae for no observed serious adverse events with 10 subjects yields a 95% confidence interval of 0–31%.

Nominal data (proportion of adverse events, proportion meeting immunological responder definitions) were analyzed by Pearson’s Chi-Square test (or Fisher’s exact test) to compare across dose levels. Antibody titers were log_10_-transformed for analysis. Between cohorts, comparisons were examined using analysis of variance (ANOVA) and over time using repeated measures ANOVA. Only subjects who received at least two doses were included in the immunologic analyses. All subjects who received at least one dose were included in the safety analyses. All analyses were performed using SAS v9.4 (Cary, NC, USA) and JMP v12, and statistical comparisons were interpreted using a two-sided alpha = 0.05.

## 3. Results

### 3.1. Demographics

**Figure 1:**
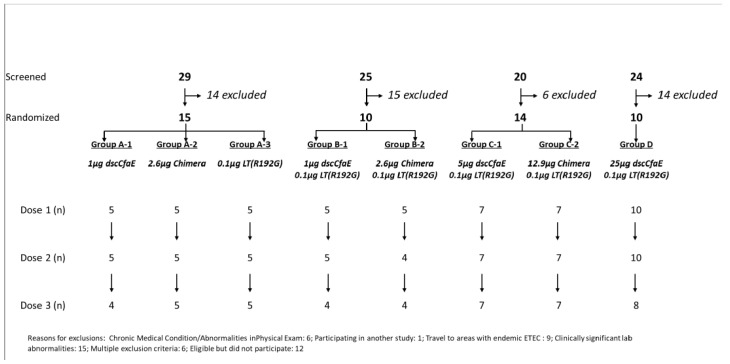
Study subject enrollment flow diagram. Footnote: Reasons for exclusion: chronic medical condition and/or abnormalities on physical exam (n = 6); participation in another study (n = 1); travel to ETEC endemic area (n = 9); clinically significant lab abnormalities (n = 15); multiple exclusion criteria (n = 6); eligible but did not participate (n = 12).

A total of 98 subjects signed a written informed consent and underwent pre-study screening for vaccination via ID or TCI route. Reasons for exclusion included abnormal medical history or physical exam (n = 6, 12.2%), recent travel to ETEC endemic region (n = 9, 18.4%), clinically significant, persistent abnormal baseline biochemistry or hematology tests (n = 15, 30.6%), and for other post-screening reasons (n = 19, 38.8%). The remaining 49 participants received at least one ID dose of study vaccine (Figure 1, Table 2). Five subjects were withdrawn prior to completion of the full vaccination series (one subject had received only one dose of vaccine and was thus excluded from immunological assessments, the remaining four subjects received at least two doses of vaccine and were included in immunological assessments). The median age of participants was 31.4 (interquartile range: 27.6, 38.9); 57.1% were male and 49.0% were Caucasian. Subjects were comparable across demographic characteristics (Table 2).

### 3.2. Safety and Clinical Adverse Events

There were no serious adverse events or adverse events that met the stopping criteria. A total of 351 adverse events were recorded of which 172 (49.0%) were defined as related (probable, possible, definite) to the vaccine (Table 3). The majority of the vaccine-related adverse events were mild and self-limited, resolving within 72 h after immunization and did not result in a change in the vaccination schedule of any subject. One subject experienced severe vaccine site pruritus. No volunteers reported any vaccine-attributable serious adverse events.

The appearance of local vaccine site reactions is described by cohort (Table 4). Every subject, except those receiving dscCfaE alone, experienced some form of vaccine site reaction (45/49, 91.8%) which included erythema (n = 45/45; 100%) and developed within 24 h of initial dosing. These initial areas of erythema resolved within 24–48 h and were followed by induration (n = 45/45; 100%), which was usually more notable at the day 7 visit. Vaccine site pain and tenderness were common and generally mild, and they were somewhat more frequent at the higher dose levels. Small, <1 cm areas of hyperpigmentation commonly followed after resolution of erythema and were seen in over 80% subjects in all groups with the exception of those who received dscCfaE alone. Edema was also noted with groups that received dscCfaE but was more prominent in groups receiving the 5 or 25 µg doses of dscCfaE. Pruritus was reported from a majority of subjects in all vaccine groups. Of note, erythema and edema at the vaccination site appeared to increase in severity and had a more rapid onset and offset at the third vaccination at all dose groups.

Among subjects in cohorts C and D, three subjects from cohort C and two subjects cohort D underwent punch biopsies at areas of hyperpigmentation at the first vaccination site. To assess the nature of hyperpigmentation changes, a protocol modification was submitted and approved to obtain punch biopsies at a limited number of vaccination sites on approximately day 70. Histology from the three subjects in cohort C (two of which received dscCfaE and one which received Chimera) revealed similar chronic inflammatory infiltrates in the dermal layers consistent with mild dermal hypersensitivity reaction and/or post-inflammatory pigmentation alteration with the presence of lymphocytes, histiocytes and melanophages; in one subject, eosinophils were also present. The pattern of infiltrates was consistent with mild dermal hypersensitivity reaction and melanosis.

### 3.3. Immunological Response

**Figure 2:**
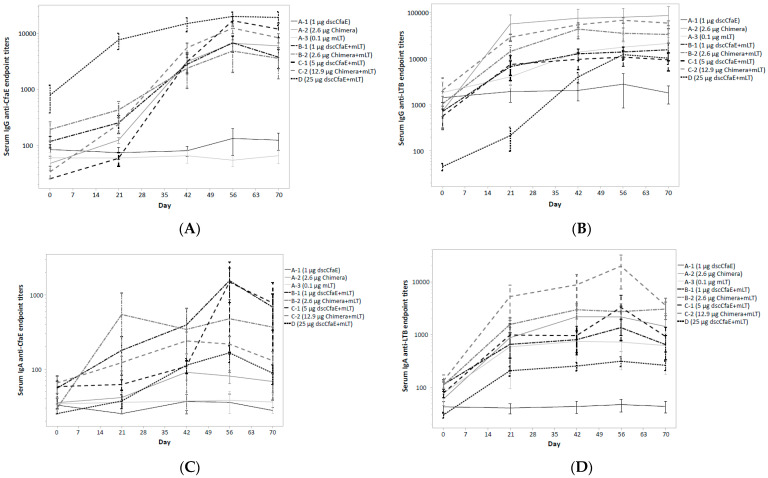
Serologic responses to CfaE (2A: IgG; 2B: IgA) and LT (2C: IgG; 2D: IgA) by study group throughout the vaccination and follow-up period. Serum IgG responses to CfaE (**A**); serum IgG responses to LT (**B**); serum IgA responses to CfaE (**C**); serum IgA responses to LT (**D**). Footnote: Endpoint titers are presented on a log scale as the mean with standard error. Subjects were vaccinated on study days 0, 21 and 42 and only those subjects receiving at least two vaccine doses are included.

The frequency of anti-dscCfaE and -toxin responses by group was assessed and is summarized in Table 5. Anti-dscCfaE IgG seroconversion was seen in all subjects receiving chimera or dscCfaE with mLT. None of the subjects receiving 1 µg of dscCfaE alone demonstrated a serum IgG response to dscCfaE (0/5; 0.0%) compared to an 80.0% (4/5) anti-dscCfaE IgG seroconversion rate in subjects receiving 1 µg of dscCfaE with 0.1 µg of mLT. With the exception of the dscCfaE-alone and mLT-alone groups (A-1 and A-3, respectively), serum anti-dscCfaE IgG Ab titers increased with each subsequent vaccination across the vaccination schedule (Figure 2A). The mean endpoint titers increased after the first dose of vaccine (Day 21) relative to baseline as well as after the second and third dose in all vaccination groups except the dscCfaE-alone and mLT-alone groups, for which there was no observable response. Maximum anti-dscCfaE IgG Ab titers were comparable across all groups receiving Chimera or dscCfaE co-administered with mLT (ANOVA *p* = 0.13). Anti-dscCfaE IgA Ab response rates were comparable across all subjects receiving either form of CfaE (dscCfaE or Chimera) co-administered with mLT (ANOVA *p* = 0.32); however, titers were less consistent over the vaccination series (Figure 2C). Subjects receiving the Chimera developed maximum IgA titers earlier, after the first or second vaccination, while subjects receiving the dscCfaE plus mLT developed maximum IgA titers following the third vaccination.

Anti-toxin IgG and IgA Ab seroconversion was seen in the majority of subjects who received mLT and/or Chimera. Antibody levels appeared to increase significantly following the first and second doses with no appreciable increases following the third dose (Figure 2B,D). Subjects receiving Chimera plus mLT had higher LTB-specific IgG and IgA antibody responses compared to subjects receiving dscCfaE + mLT (*t*-test *p* < 0.001).

**Figure 3:**
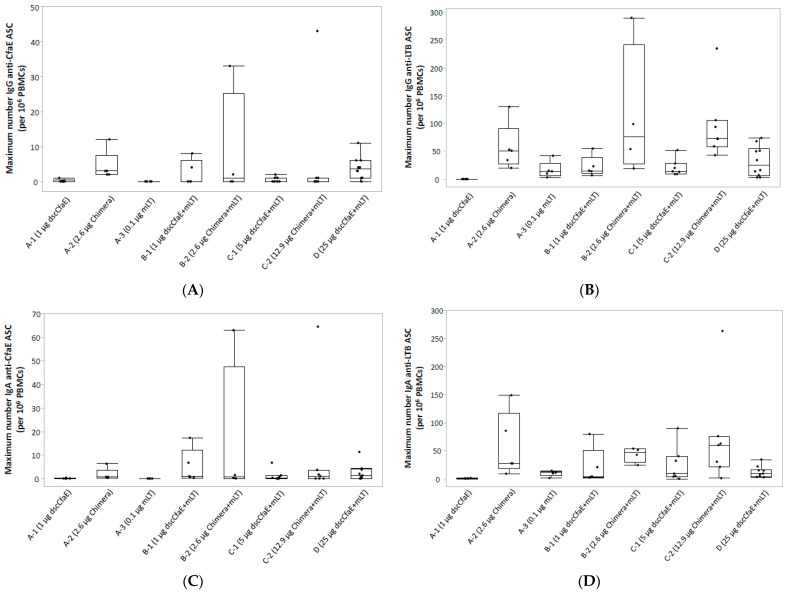
Maximum number of post-vaccination antibody-secreting cells (ASCs) to CfaE (3A: IgG; 3B: IgA) and LT (3C: IgG; 3D: IgA) per 10^6^ peripheral blood mononuclear cells (PBMCs). IgG ASC responses to CfaE (**A**); IgG ASC responses to LT (**B**); IgA ASC responses to CfaE (**C**); IgA ASC responses to LT (**D**). Footnote: The maximum number of antibody-secreting cells (ASCs) post-vaccination is reported with accompanying box and whisker plots where the mid-line in the box represents the median, the top and bottom of the box represent the 1st and 3rd quartiles, respectively, and the whiskers extend to 1.5xIQR (interquartile range). Subjects were vaccinated on study days 0, 21 and 42, and only those subjects receiving at least two vaccine doses are included. ASC samples were collected at baseline and seven days after each vaccination.

IgG and IgA ASC responses to dscCfaE were only present among groups that received Chimera or dscCfaE in combination with mLT (Figure 3A and Figure 3C, respectively), highlighting an adjuvant effect when mLT was co-administered with dscCfaE but less apparent when co-administered with Chimera; however, there was no significant difference in the maximum number of ASCs across groups receiving dscCfaE or Chimera regardless of dose or adjuvant (IgA: Wilcoxon *p* = 0.2). Anti-toxin IgG and IgA ASC responses (Figure 3B and Figure 3D, respectively) were present among most groups receiving Chimera or mLT. There was no significant difference in the maximum number of anti-toxin IgA ASCs across those groups (Wilcoxon *p* = 0.06); however, subjects receiving Chimera with mLT had a significantly higher number of anti-toxin IgG ASCs than subjects receiving dscCfaE with mLT (Wilcoxon *p* = 0.0002).

**Figure 4:**
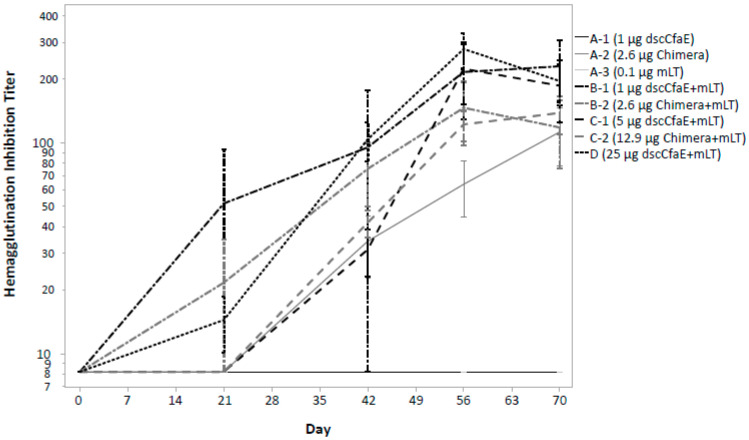
Hemagglutination inhibition (HAI) from serum samples by study group throughout the vaccination and follow-up period. Footnote: Each sample was tested in duplicate, and the HAI titer was expressed as the average of the reciprocal of the highest serum dilution that completely inhibited MRHA. Data are presented on a log scale as the mean with standard error. Subjects were vaccinated on study days 0, 21 and 42 and only those subjects receiving at least two vaccine doses are included.

Hemagglutination inhibition was detected in all groups except those who received either dscCfaE or mLT alone (Figure 4—cohort groups A-1 and A-3). Maximal HAI responses peaked following the third vaccination in group A-2 and all subjects in cohorts B and C with little difference in the magnitude of the responses among those groups.

## 4. Discussion

This study represents the first intradermal administration of dscCfaE or mLT in humans and the first administration of the Chimera. The data indicate that both dscCfaE and the Chimera are safe and immunogenic after ID administration with mLT. We confirmed the preclinical observation that indicated the capacity of mLT to enhance the immune response to a co-administered antigen, i.e., dscCfaE. There were no limiting systemic vaccine reactions. The most common adverse events were local vaccine site reactions, which were seen in the majority of subjects (91.23%). Adverse reactions were predominantly mild and brief. Vaccine site reactions were characterized most often by erythema followed by induration which was noted in all groups other than those who received dscCfaE alone. Small areas of hyperpigmentation at the vaccination site were common upon the resolution of erythema. Pruritus and local vaccine tenderness and pain were also common. As such, we noted reactogenicity at all dose levels with the notable exception among subjects who received low-dose dscCfaE without mLT. The histopathology findings from the limited number of subjects on whom we performed punch biopsy evaluation of the first vaccination sites suggest some residual chronic inflammation with dermal and epidermal hyperpigmentation. The findings were non-specific and suggested residual evidence of an immune response to the administered antigens. Our findings and rates of induration and tenderness have also been seen in published ID vaccination studies with other antigens, and they may be due in part to factors associated with the cutaneous vaccination route as well as reactogenicity to the antigens [39,42,44].

In addition to no dose-limiting safety concerns, ID administration of these antigens induced robust immunologic responses. Across all cohorts receiving either the Chimera or dscCfaE co-administered with mLT, rates of serum IgG and IgAAb seroconversion rates to dscCfaE and toxin were high. The magnitude and kinetics of the serum responses were also robust with both dscCfaE and Chimera eliciting brisk rises in anti-dscCfaE, which generally peaked after the third dose and were notable at the low- and high-dose groups of dscCfaE and Chimera. In terms of primary cellular immune responses, by quantification of ASCs, anti-dscCfaE responses seen after vaccination with Chimera and with the high-dose dscCfaE, whereas anti-toxin responses were seen among all subjects who received mLT in combination with dscCfaE or Chimera. Results from functional assays performed by HAI with CFA/I^+^ ETEC were notable for the presence of neutralizing antibodies after the second dose among most groups that received dscCfaE or Chimera plus mLT and after the third dose for those receiving Chimera alone.

The first of two CfaE-based antigens which our team advanced to human trials via cutaneous routes was dscCfaE, which was initially administered in an open-label, dose-escalating study by TCI vaccination with and without a fixed dose of mLT [29]. While anti-toxin responses were observed, anti-dscCfaE immune responses were minimal by the TCI route. Our findings reveal encouragingly robust immune responses and a clear adjuvant effect of mLT when administering with these antigens by the ID route. The different immune responses observed in trials may be due to differential antigen penetration or product dissociation between antigen and adjuvant when delivered by TCI. With ID delivery, ASC and HAI responses were robust across the dosing schedules. Reactogenicity was acceptable and defined by expected and well-tolerated mild local vaccine site reactions. Further evaluation of the immune responses is underway to include fecal and salivary immune assays as well as other assays to evaluate serum and cellular immune responses.

While no anti-dscCfaE responses were observed with 1 μg of dscCfaE alone, the same dose given with mLT led to anti-dscCfaE IgG and IgA Ab seroconversion, demonstrating the adjuvant effect of attenuated toxin. In parallel, mLT was also a potent antigen eliciting robust serologic and cellular anti-toxin responses even when administered at the low dose of 0.1 μg. Although the transient local reactogenicity observed after the intradermal administration of mLT was anticipated in preclinical studies in mice, it is possible that a further attenuated molecule, the double mutant of LT (LT(R192G/L211A) or dmLT), can offer similar adjuvant capacity with diminished reactogenicity [45,46,47]. In fact, dmLT has recently been successfully evaluated in different clinical trials [30,46,47,48].

In summary, both CfaE-based vaccine candidates, dscCfaE and Chimera, induced serologic responses in a dose–response manner, and the inclusion of mLT increased the functional responses. After a review of the safety and immunogenicity data available, intradermally administered dscCfaE at 25 µg, co-administered with mLT, was selected for advancement to a Phase 2b vaccination-challenge trial to evaluate protective efficacy against CFA/I^+^ ETEC strain H10407 in a controlled human infection model (CHIM) (ClinTrials.gov Identifier: NCT01922856).

## Figures and Tables

**Table 1:** Phase 1 clinical trial design group allocation.

Group	n	dscCfaE	Chimera	mLT
A-1	5	1 µg	--	--
A-2	5	--	2.6 µg	--
A-3	5	--	--	0.1 µg
B-1	5	1 µg	--	0.1 µg
B-2	5	--	2.6 µg	0.1 µg
C-1	7	5 µg	--	0.1 µg
C-2	7	--	12.9 µg	0.1 µg
D	10	25 µg	--	0.1 µg

**Table 2:** Demographic features by study group A–D.

Cohort	A-1 (N = 5)	A-2 (N = 5)	A-3 (N = 5)	B-1 (N = 5)	B-2 (N = 5)	C-1 (N = 7)	C-2 (N = 7)	D (N = 10)
**Median Age (IQR)**	28.4 (24.8, 30.3)	31.0 (26.6, 33.4)	31.2 (26.3, 32.6)	31.9 (31.7, 42.5)	38.9 (33.8, 42.2)	32.5 (25.9, 34.2)	30.2 (25.5, 32.3)	30.5 (27.6, 42.7)
**Gender**								
Female, n (%)	0 (0.0%)	1 (20.0%)	2 (40.0%)	0 (0.0%)	2 (40.0%)	5 (71.4%)	5 (71.4%)	6 (60.0%)
Male, n (%)	5 (100%)	4 (80.0%)	3 (60.0%)	5 (100%)	3 (60.0%)	2 (28.6%)	2 (28.6%)	4 (40.0%)
**Race**								
Black, n (%)	2 (40.0%)	3 (60.0%)	1 (20.0%)	2 (40.0%)	3 (60.0%)	1 (14.3%)	3 (42.9%)	5 (50.0%)
Caucasian, n (%)	3 (60.0%)	1 (20.0%)	4 (80.0%)	2 (40.0%)	2 (40.0%)	5 (71.4%)	3 (42.9%)	4 (40.0%)
American Indian, n (%)	0 (0.0%)	1 (20.0%)	0 (0.0%)	0 (0.0%)	0 (0.0%)	0 (0.0%)	0 (0.0%)	0 (0.0%)
Asian, n (%)	0 (0.0%)	0 (0.0%)	0 (0.0%)	1 (20.0%)	0 (0.0%)	0 (0.0%)	0 (0.0%)	1 (10.0%)
Other, n (%)	0 (0.0%)	0 (0.0%)	0 (0.0%)	0 (0.0%)	0 (0.0%)	1 (14.3%)	1 (14.3%)	0 (0.0%)
**Ethnicity, n (%)**								
Hispanic/Latino, n (%)	1 (20.0%)	1 (20.0%)	0 (0.0%)	0 (0.0%)	0 (0.0%)	1 (14.3%)	0 (0.0%)	0 (0.0%)

**Table 3:** Frequency of adverse events coded as related* to the investigational products.

AE	Mild N (%)	Moderate N (%)	Severe N (%)	Total N (%)
Arthralgia	1 (2.0)	0 (0.0)	0 (0.0)	1 (2.0)
Axillary Pain	1 (2.0)	0 (0.0)	0 (0.0)	1 (2.0)
Fasciculation	1 (2.0)	0 (0.0)	0 (0.0)	1 (2.0)
Fever	2 (4.1)	0 (0.0)	0 (0.0)	2 (4.1)
GI Symptoms	0 (0.0)	1 (2.0)	0 (0.0)	1 (2.0)
Headache	6 (12.2)	1 (2.0)	0 (0.0)	7 (14.3)
Hemoglobin Decrease	4 (8.2)	1 (2.0)	0 (0.0)	5 (10.2)
Knee Pain	1 (2.0)	0 (0.0)	0 (0.0)	1 (2.0)
Leukopenia	1 (2.0)	0 (0.0)	0 (0.0)	1 (2.0)
Loose Stool	5 (10.2)	0 (0.0)	0 (0.0)	5 (10.2)
Lymphadenopathy	1 (2.0)	0 (0.0)	0 (0.0)	1 (2.0)
Malaise	1 (2.0)	2 (4.1)	0 (0.0)	3 (6.1)
Myalgia	2 (4.1)	0 (0.0)	0 (0.0)	2 (4.1)
Sleepiness	0 (0.0)	1 (2.0)	0 (0.0)	1 (2.0)
Thrombocytopenia	1 (2.0)	0 (0.0)	0 (0.0)	1 (2.0)
Vaccination Site Pain	19 (38.8)	2 (4.1)	0 (0.0)	21(42.9)
Vaccination Site Pruritus	32 (65.3)	3 (6.1)	1 (2.0)	36 (73.5)
Vaccine Site Reaction	44 (89.8)	1 (2.0)	0 (0.0)	45 (91.8)
Vaccine Site Swelling	4 (8.2)	0 (0.0)	0 (0.0)	4 (8.2)
Vaccine Site Tenderness	32 (65.3)	1 (2.0)	0 (0.0)	33 (67.3)

Footnote: Includes all subjects who received at least one vaccine dose and limited to adverse events coded as either definitely, probably or possibly related to the investigational products.

**Table 4:** Description of local vaccine site reactions following intradermal vaccination with CfaE, Chimera, mLT alone or CfaE and Chimera co-administered with mLT.

Group	Cohort A-1 CfaE 1 µg	Cohort A-2 Chimera 2.6 µg	Cohort A-3 mLT 0.1 µg	Cohort B-1 CfaE 1 µg (+)mLT	Cohort B-2 Chimera 2.6 µg (+)mLT	Cohort C-1 CfaE 5 µg (+)mLT	Cohort C-2 Chimera 12.9 µg (+)mLT	Cohort D CfaE 25 µg (+)mLT
N	5	5	5	5	5	7	7	10
Pain	0 (0.0)	2 (40.0)	1 (20.0)	1 (20.0)	3 (60.0)	4 (57.1)	5 (71.4)	5 (50.0)
Pruritus	0 (0.0)	4 (80.0)	4 (80.0)	4 (80.0)	5 (100)	5 (71.4)	6 (85.7)	8 (80.0)
Tenderness	1 (20.0)	3 (60.0)	3 (60.0)	3 (60.0)	3 (60.0)	6 (85.7)	6 (85.7)	8 (80.0)
Vaccine Site Reaction	1 (20.0)	5 (100)	5 (100)	5 (100)	5 (100)	7 (100.0)	7 (100)	10 (100)
Erythema	1 (20.0)	5 (100)	5 (100)	5 (100)	5 (100)	7 (100.0)	7 (100)	10 (100)
Induration	0 (0.0)	5 (100)	5 (100)	5 (100)	5 (100)	7 (100.0)	7 (100)	10 (100)
Papules	0 (0.0)	2 (40.0)	0 (0.0)	0 (0.0)	0 (0.0)	0 (0.0)	0 (0.0)	2 (20.0)
Edema	0 (0.0)	2 (40.0)	1 (20.0)	3 (60.0)	4 (80.0)	5 (71.4)	7 (100)	7 (70.0)
Hypopigmentation	0 (0.0)	2 (40.0)	1 (20.0)	0 (0.0)	1 (20.0)	0 (0.0)	0 (0.0)	0 (0.0)
Hyperpigmentation	0 (0.0)	4 (80.0)	5 (100)	5 (100)	5 (100)	6 (85.7)	7 (100)	8 (80.0)
Vesicles	0 (0.0)	0 (0.0)	1 (20.0)	0 (0.0)	0 (0.0)	0 (0.0)	0 (0.0)	0 (0.0)

Footnote: Includes all subjects who received at least one vaccine dose and limited to local vaccine site reactions. mLT was co-administered at a 0.1 µg dose.

**Table 5:** Frequency of responses across immunologic assays and antigens.

Group		N	Serology				ASC	
			IgG		IgA		IgA	
			CfaE	LTB	CfaE	LTB	CfaE	LTB
A	1 µg dscCfaE	5	0 (0.0)	0 (0.0)	0 (0.0)	0 (0.0)	0 (0.0)	2 (40.0)
	2.6 µg Chimera	5	5 (100)	5 (100)	1 (20.0)	4 (80.0)	1 (20.0)	5 (100)
	0.1 µg mLT	5	0 (0.0)	5 (100)	0 (0.0)	3 (60.0)	0 (0.0)	5 (100)
B	1 µg dscCfaE + 0.1 µg mLT	5	4 (80.0)	5 (100)	3 (60.0)	4 (80.0)	3 (60.0)	5 (100)
	2.6 µg Chimera + 0.1 µg mLT	4	4 (100)	4 (100)	2 (50.0)	4 (100)	2 (50.0)	4 (100)
C	5 µg dscCfaE + 0.1 µg mLT	7	7 (100)	7 (100)	3 (42.9)	5 (71.4)	2 (28.6)	6 (85.7)
	12.9 µg Chimera + 0.1 µg mLT	7	7 (100)	7 (100)	3 (42.9)	7 (100)	4 (57.1)	7 (100)
D	25 µg dscCfaE + 0.1 µg mLT	10	9 (90.0)	9 (90.0)	5 (50.0)	7 (70.0)	6 (60.0)	10 (100)

Immunologic responses are limited to subjects receiving at least two vaccine doses. Serologic responses were identified as a ≥4-fold increase over baseline titer, and an ASC response was defined as a ≥2-fold increase over the baseline number of ASCs per 10^6^ PBMCs or post-vaccination value > 1 ASC per 10^6^ PBMCs if the baseline number of cells was 0 per 10^6^ PBMCs. The adjuvant LT(R192G) is abbreviated as mLT.

## Data Availability

Data are contained within the article. The article will be published in a fully open access journal to help ensure widespread data dissemination.
